# Exploring waste separation using an extended theory of planned behavior: a comparison between adults and children

**DOI:** 10.3389/fpsyg.2024.1337969

**Published:** 2024-04-18

**Authors:** Ji Pan, Pingping Liu

**Affiliations:** ^1^CAS Key Laboratory of Mental Health, Institution of Psychology, Chinese Academy of Sciences, Beijing, China; ^2^Department of Psychology, University of Chinese Academy of Sciences, Beijing, China

**Keywords:** environmental knowledge, environmental awareness, waste separation, theory of planned behavior, children

## Abstract

This study applied an extended model of the theory of planed behavior (TPB) to compare the differences in waste separation behavior between children (ages 9 to 12, *N* = 339) and adults (ages 18 to 66, *N* = 379). We examined the relations among waste separation attitude, subjective norm, perceived behavioral control, knowledge, awareness, intention, and behavior. The results showed waste separation knowledge of children was less than that of adults. Structure equation model results also revealed robust differences between children and adults. For adults, TPB variables (attitude, subjective norm, and perceived behavioral control) and knowledge are significantly positively related to their waste separation intention. Meanwhile, perceived behavioral control and intention are positively related to adults’ behavior. However, for children, only perceived behavioral control and awareness are positively related to intention, and perceived behavioral control is positively related to behavior. Moreover, the predictive power of the extended TPB model on children’s waste separation intention and behavior are lower than those of adults. The different results may be due to children’s immature cognitive abilities. This study enhanced the understanding of the different waste separation behavior determinants between children and adults. The findings are useful for developing tailored policies and promoting children’s waste separation behavior.

## 1 Introduction

Children are one of the most important agencies of waste separation. They are also potential future leaders and can bring robust social impacts to people around them (Meinhold and Malkus, 2005; Liao and Li, 2019; Deng et al., 2022; Wang et al., 2022). Moreover, children’s environmental attitude and behavior today can directly affect their future attitude and behavior (Evans et al., 2007; Arı and Yılmaz, 2017; Otto et al., 2019). Therefore, it is necessary to develop tailored promoting strategies for children. Accordingly, it is essential to explore the determinants of children’s waste separation behavior, and find these aged-related differences between children and adults.

Many studies have explored the factors influencing adults’ waste separation behavior. For instance, attitude (Wang, 2021), perceived behavioral control (Shi et al., 2021; Wang et al., 2021; Lou et al., 2022), intention (Shi et al., 2021; Wang, 2021), contextual factors (Fan et al., 2019; Meng, 2019; Leeabai et al., 2021), habitual factors (Fan et al., 2019), knowledge (Meng, 2019), and incentive measures (Wang et al., 2020) play positive roles in waste separation behavior. However, there is relatively little research on children’s waste separation behavior.

Prior studies on primary students’ environmental behavior mainly focus on general environmental behavior (Evans et al., 2007; Chawla and Derr, 2012; Collado et al., 2015; Otto and Pensini, 2017), sustainable behavior (Tucker and Izadpanahi, 2017; Ebersbach and Brandenburger, 2020), recycling behavior (Kumar, 2019; Šorytė and Pakalniškienė, 2021), and food waste (Niaki et al., 2017; Sorokowska et al., 2020), but neglect waste separation behavior. What are the main factors influencing children’s waste separation behavior, and are these factors different from those for adults? To narrow the research gap, this study focused on children’s waste separation behavior and made a comparison with adults by using the theory of planned behavior (TPB; Ajzen, 1991).

TPB is one of the most influential models for exploring the factors affecting people’s behavior (Yuriev et al., 2020), and is widely used in research on environmental behavior (Yu et al., 2018; Meng, 2019). However, to our knowledge, applications of extended TPB to children’s waste separation behavior are scarce. Moreover, since TPB has some limitations due to its incompleteness (de Leeuw et al., 2015; Yuriev et al., 2020), researchers have expanded TPB by adding other factors to enhance its predictive power, such as knowledge (Pothitou et al., 2016; Liao and Li, 2019; Xie and Lu, 2022), social norms (Goh et al., 2022; Guo et al., 2022), descriptive norms (Qalati et al., 2022), moral norms (Wan et al., 2017), moral responsibility (Qalati et al., 2022), and awareness of the consequences (Wan et al., 2017; Kumar, 2019; Meng, 2019; Zhang et al., 2019). Knowledge is an important prerequisite for action (Otto and Pensini, 2017), and awareness is recognized as the important antecedent of behavior (Fu et al., 2020). Therefore, we integrate knowledge and awareness to TPB, and attempt to answer the two key questions: (1) Could children’s waste separation behavior be explained by the expanded model of TPB? (2) Is there any difference in the influencing factors between children’s and adults’ waste separation behavior?

The present study provides three main contributions. First, it tests the determinants of children’s waste separation behavior. Second, it explores the differences in waste separation between children and adults. Third, it extends TPB by adding two additional variables (i.e., knowledge and awareness) to better understand children’s and adults’ waste separation intention and habit formation processes.

## 2 Literature review and the conceptual model

### 2.1 The theory of planed behavior

The theory of planned behavior (TPB) proposed by Ajzen (1991) is a popular and validated social-cognitive model of human behavior in specific contexts. According to TPB, attitude, subjective norm, and perceived behavioral control can directly influence intention, and intention can directly influence behavior. Perceived behavioral control can also directly promote behavior (Ajzen, 1991). The predictive power of TPB for adults’ environmental intention and behavior has been widely demonstrated (de Leeuw et al., 2015; Ma et al., 2018; Liao and Li, 2019; Šorytė and Pakalniškienė, 2021; Qalati et al., 2022), such as recycling (Wan et al., 2021), energy saving (Alzubaidi et al., 2021; Qalati et al., 2022), sustainable development (Blok et al., 2015), and waste separation (Goh et al., 2022).

However, there is a paucity of studies using TPB to explore children’s behavior (Šorytė and Pakalniškienė, 2021). Meanwhile, the conclusions of few studies using TPB to explore children’s behavior remain controversial. Šorytė and Pakalniškienė (2021) found when using the original TPB model, affective attitude and perceived behavioral control have a significant effect on children’s (ages 8 to 11) intention to recycle, and perceived behavioral control and intention have a significant effect on their recycling behavior. However, after introducing three variables including parental behavior, gender, and social desirability to the model, intention was not a significant antecedent of recycling behavior, and perceived behavioral control was not a significant antecedent of intention to recycle. Wang and Wang (2015) found that intention has a significant effect on children’s (ages 9 to 13) physical activity, attitude and perceived behavioral control have a significant effect on intention to engage in physical activity while subjective norm not. Jeon and Cha (2019) found that intention can significantly affect fifth and sixth-grade children’s health promotion behavior, and subjective norm and perceived behavioral control can significantly affect the health promotion intention while attitude can not.

To further understand the factors influencing children’s waste separation behavior, we propose the following hypotheses related to TPB:

*H1.1*: Attitude has a positive effect on children’s waste separation intention.

*H1.2*: Subjective norm has a positive effect on children’s waste separation intention.

*H1.3*: Perceived behavioral control has a positive effect on children’s waste separation intention.

*H1.4*: Perceived behavioral control has a positive effect on children’s waste separation behavior.

*H1.5*: Intention has a positive effect on children’s waste separation behavior.

### 2.2 The extended variables: environmental knowledge and awareness

With no doubt, TPB provides a useful framework to explore the mechanism of individual behavior change. However, due to the original TPB model’s limitation, numerous studies extended the model to improve its predictive power (Pothitou et al., 2016; Wan et al., 2017; Kumar, 2019; Liao and Li, 2019; Meng, 2019; Zhang et al., 2019; Goh et al., 2022; Guo et al., 2022; Xie and Lu, 2022). Knowledge and awareness are two of the most frequent variables included in the extended TPB model (Yuriev et al., 2020).

Environmental knowledge is widely used in the literature to predict pro-environmental behavior. Although Knowledge seems to be a necessary prerequisite to performing environmental behavior (Otto and Pensini, 2017), the empirical evidence is inconclusive. On one hand, many studies have shown that knowledge can affect environmental behavior significantly (Meinhold and Malkus, 2005; Pagiaslis and Krontalis, 2014; Pothitou et al., 2016; Casaló et al., 2019; Liao and Li, 2019; Saari et al., 2021; Wu et al., 2022). According to Meinhold and Malkus (2005), when attitude was controlled, knowledge has a significant positively influence on adolescents’ environmental behavior. Wu et al. (2022) noted that knowledge can improve college students’ perceived behavioral control and environmental attitude, thus promoting the implementation of environmental behaviors. Conversely, some studies also showed that knowledge may not necessarily influence environmental behavior (Grob, 1995; de Leeuw et al., 2015; Otto et al., 2015; Otto and Pensini, 2017). de Leeuw et al. (2015) mentioned it is not enough to change behavior patterns only through transmitting knowledge. Otto et al. (2015) also found that when controlling the income variable, there was no significant correlation between environmental knowledge and behavior.

The impact of awareness on environmental behavior remains controversial. Some studies suggested that awareness positively affects people’s environmental behavior (Gan et al., 2021; Rotaris et al., 2021; Soares et al., 2021). On the contrary, Fu et al. (2020) found that there is an environmental awareness-behavior gap, and perceived effectiveness of policy can fill the gap. Liobikienė and Juknys (2016) found that the lack of knowledge or information may limit the influence that awareness made on behavior.

To advance the research on the role of knowledge and awareness in environmental intention and behavior, the present study adopts a series of objective and verifiable questions to assess participants’ objective knowledge. Awareness is defined as the perception of the consequence of environmental issues. We integrated the two extended variables into TPB model and propose the following hypotheses:

*H2*: Knowledge has a positive effect on children’s waste separation intention.

*H3*: Awareness has a positive effect on children’s waste separation intention.

### 2.3 TPB and the development of children’s cognitive ability

It is worth noting that cognitive ability plays an important part in TPB (Ajzen, 1991). Intention, the important mediator in TPB, depends on the cognitive ability (Gazzaniga and Heatherton, 2006). Meanwhile, children’s cognitive ability is still developing. Their decision-making ability based on their intention is also developing (Gazzaniga and Heatherton, 2006; Wang et al., 2022).

When acting health behaviors, for example, older groups are driven by more rational considerations when compared to younger groups (Roux et al., 2021). Same for environmental issues, older groups have a more mature cognitive ability to understand such problems (Collado et al., 2015). That’s why some researchers argued that the model was more suitable for older groups (Lee et al., 2021). Therefore, we propose the following hypotheses:

*H4*: The predictive power of TPB on children’s waste separation intention and behavior is smaller than on adults.

**Figure 1:**
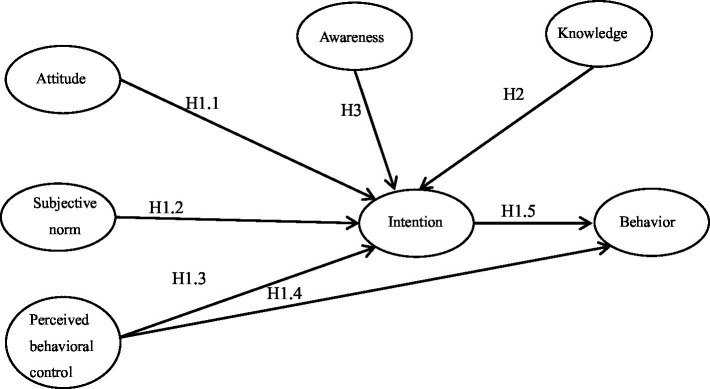
Conceptual model for both children and adults.

The hypothesized model is illustrated in Figure 1.

## 3 Method

### 3.1 Measurement

In the first section of the questionnaire, we measured attitude, subjective norm, perceived behavioral control, waste separation awareness, waste separation intention, and waste separation behavior (see Table 1). To ensure the reliability and validity of each variable, all items were designed based on the literature and were modified to meet the current study context (Zhang et al., 2019). All measurement items were on a seven-point scale ranging from 1 to 7. For behavior, 1 stands for “Never,” and 7 stands for “Always.” For the rest variables (attitude, subjective norm, perceived behavioral control, waste separation awareness, and waste separation intention), 1 stands for “Strongly disagree,” 7 stands for “Strongly agree.”

**Table 1:** Description of measurement items and factor loading.

Variable	Measurement items	Factor loading	
		Children	Adults
Attitude			
1	I think it is good to separate garbage	0.765	0.879
2	I think it is important to separate garbage to protect the environment	0.838	0.918
3	I think waste separation is valuable in alleviating the shortage of nature resources		0.763
Subjective norm			
1	My family (parents) would think I should separate the garbage	0.780	0.870
2	My friends would think I should separate the garbage	0.777	0.914
3	My colleagues (classmates) would think I should separate the garbage	0.811	0.869
Perceived behavioral control			
1	I feel inconvenient when separate the waste in my daily life	0.590	0.779
2	It is not up to me whether or not I separate the garbage	0.892	0.763
3	I do not know how to separate the garbage	0.766	0.615
Awareness			
1	Failure to separate the garbage will cause pollution	0.683	0.791
2	Failure to separate the garbage can lead to over-consumption of nature resources	0.818	0.928
3	Failure to separate the garbage can have a pernicious effect on the life of descendants	0.782	0.881
Intention			
1	I will separate recyclables when I put out the garbage	0.693	0.813
2	I will separate food waste when I put out the garbage	0.475	0.837
3	I will separate hazardous waste when I put out the garbage	0.926	0.631
4	I will separate residual waste when I put out the garbage	0.847	0.817
Behavior			
1	How often do you separate recyclables from other garbage	0.806	0.900
2	How often do you separate food waste from other garbage	0.931	0.868
3	How often do you separate dangerous waste from other garbage	0.625	0.920
4	How often do you separate residual waste from other garbage	0.722	0.710

In the second section, knowledge was assessed through twenty objective items. We adopted the questions designed by the environmental protection organization, “Friends of Nature.” There were twenty different items listed (see Table 2) and each with four options: recyclables, food waste, hazardous waste, and residual waste. The scores of knowledge were assessed through the accuracy, which was determined based on the Beijing Municipal Garbage Management Regulations. The last section was the demographic characteristics of respondents.

**Table 2:** Waste separation knowledge items.

Recyclables	Food waste	Residual waste	Hazardous waste
Screw, old mobile phone, glass bottle, plastic basin, metal bottle cap, old shoes, iron can, old towel	Tea leaves, stale bread, star anise, cinnamon or other seasonings	Broken pen, plastic bag, used tissues, cigarette butts, wet tissue	Mobile power supply (charge Pal), broken CFLS, insecticide can, broken thermometer

We distributed the questionnaire to 10 children (ages 9 to 12 years), and 9 adults, as a pilot study. Items adapted from the literature were slightly modified for consistency with the samples. According to the differences between adults and children, we changed some statements in the questions between the two groups. For example, to measure children’s subjective norm, we replaced “family” and “colleagues” with “parents” and “classmates,” respectively. Based on Cronbach’s *α* value, we removed an item from children’s attitude scale.

### 3.2 Data collection and sampling

The survey data of children was collected from two primary schools in Beijing in October 2022. We chose the two schools because their teachers, students, and parents agreed to participate in our study during the COVID-19 pandemic, and the number of students in the two schools met our research criteria. A total of 400 questionnaires were distributed to students in grades 4–6. After eliminating 21 questionnaires with unfinished items or clearly perfunctory answers, a total of 379 (197 girls, ages 9 to 12, M_age_ = 10.41 years, SD_age_ = 0.90 years) questionnaires were considered valid. We chose this stage not only because it is the earliest stage at which children can understand and answer written questions (Ando et al., 2015). More importantly, it has been confirmed the stage children start to form and consolidate their environmental attitude and behavior (Otto et al., 2019; Sorokowska et al., 2020). The data from adults were collected through Wen Juan Xing. 400 questionnaires were distributed from August to October 2022. After eliminating 61 questionnaires with unfinished items or clearly perfunctory answers, a total of 339 (241 females, ages 18 to 66, M_age_ = 37.40 years, SD_age_ = 7.27 years) were considered valid. For a model with seven variables, the minimum sample size is 150, and the preferred sample size is about 10 times the number of items (Hair et al., 2009). Thus, the valid responses of this study satisfied the minimum requirements.

The study was approved by the Institutional Review Board of the Institute of Psychology, Chinese Academy of Sciences. Introduction to the study along with an informed consent form was given to all participating children and their parents before the interventions.

### 3.3 Data analysis

SPSS 27.0 and AMOS 25.0 were used to analyze the data. First, the measurement model was tested to confirm the reliability and validity. The reliability of the scale was tested by Cronbach’s *α* and composite reliability (CR). KMO and Bartlett’s test were performed to test the structure validity. Convergent validity was tested by factor loading values and average variance extract (AVE). Discriminant validity was tested by comparing the correlation between each two variables and the square root of AVE. Second, independent samples *t*-test was used to compare the means of each variable between children and adults. Third, to test whether the expanded TPB model is applicable to explain children’s waste separation intention and behavior, and to compare the factors of children and adults, a structural equation model was built based on the conceptual model.

## 4 Results

### 4.1 Reliability and validity

Table 3 shows that Cronbach’s *α* and composite reliability (CR) of the sub-scales were all above the threshold level of 0.70, thus satisfied the minimum requirements (Fornell and Larcker, 1981). KMO and Bartlett’s test were performed to test the structure validity. The value of KMO was 0.799 and 0.850 in the children’s and adults’ scales, respectively. Bartlett’s test of Sphericity was significant (*p* < 0.01), indicated that the items were suitable for exploratory factor analysis (EFA). The results showed that 52.597% (children) and 69.431% (adults) of the total variance was explained, thus satisfied the minimum requirements (Hair et al., 1998).

**Table 3:** Means, standard deviation, Cronbach’s *α*, composite reliability, AVE, and correlation matrix of variables.

	M ± SD	*α*	CR	AVE	1	2	3	4	5	6
Children										
1 Awareness	6.777 ± 0.562	0.777	0.806	0.582	0.763					
2 Perceived behavioral control	6.761 ± 0.622	0.773	0.799	0.577	0.448^**^	0.760				
3 Subjective norm	6.810 ± 0.524	0.710	0.832	0.623	0.445^**^	0.368^**^	0.789			
4 Attitude	6.694 ± 0.194	0.741	0.783	0.644	0.383^**^	0.309^**^	0.462^**^	0.802		
5 Intention	6.826 ± 0.580	0.826	0.834	0.570	0.357^**^	0.332^**^	0.323^**^	0.229^**^	0.755	
6 Behavior	6.383 ± 0.955	0.820	0.858	0.607	0.228^**^	0.301^**^	0.309^**^	0.184^**^	0.178^**^	0.779
Adults										
1 Awareness	6.214 ± 1.046	0.901	0.902	0.754	0.868					
2 Perceived behavioral control	5.041 ± 1.480	0.758	0.765	0.522	0.342^**^	0.722				
3 Subjective norm	6.124 ± 1.091	0.913	0.915	0.783	0.651^**^	0.419^**^	0.885			
4 Attitude	6.592 ± 0.769	0.878	0.891	0.733	0.760^**^	0.286^**^	0.659^**^	0.856		
5 Intention	6.161 ± 0.962	0.850	0.859	0.607	0.622^**^	0.449^**^	0.733^**^	0.610^**^	0.779	
6 Behavior	5.752 ± 1.321	0.906	0.914	0.729	0.417^**^	0.451^**^	0.543^**^	0.399^**^	0.630^**^	0.854

**p < 0.01.

Convergent validity was tested by the value of factor loading and average variance extract (AVE). According to previous studies, factor loading exceeded 0.4, and AVE exceeded 0.5 (Chin, 1998; Hair et al., 1998), indicated that the measurement had good convergence validity. Tables 1, 3 show that the convergent validity of the scale in this study was acceptable.

Discriminant validity was tested by comparing the correlation between each two variables and the square root of AVE (Goodhue, 1998). As Table 3 showed, the values on the diagonal line were the square roots of AVE, while other values were the correlation between each two variables. The square roots of AVE were all higher than the correlation with any of the other constructs, thus meeting the criteria required to establish discriminant validity (Fornell and Larcker, 1981).

### 4.2 Means

**Figure 2:**
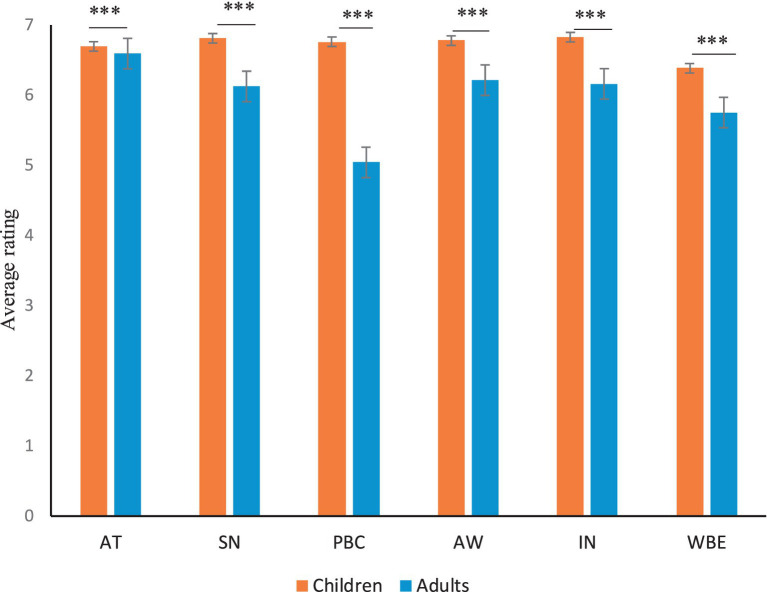
Means of the TPB variables and awareness. AT, attitude; SN, subjective norm; PBC, perceived behavioral control; AW, waste separation awareness; IN, waste separation intention; WBE, waste separation behavior. ***p < 0.001. Error bars represent standards errors of the mean.

Independent samples *t*-test was used to compare the mean values of each variable between children and adults. Figure 2 shows that there were significant differences between the two groups (*p* < 0.001). Children’s waste separation attitude, subjective norm, perceived behavioral control, waste separation awareness, intention and behavior, were significantly higher than those of adults (*p* < 0.001). Nevertheless, the accuracy of children’s waste separation knowledge (69.3%), which was tested through objective questions, was significantly lower than that of adults (76.6%; *p* < 0.001). Meanwhile, a discrepancy was found between waste separation intention and behavior in both the two groups. That is, the mean values of behavior were both lower than the mean values of intention.

### 4.3 Testing the expanded TPB model

To test whether the expanded TPB model is applicable to explain children’s waste separation intention and behavior, and to compare the determinants of children and adults, a structural equation model was built based on the conceptual model. Amos 25.0 was used to assess the overall model fit, coefficient, and significance of the structural paths. Table 4 showed the fit indices indicated that the model provided an excellent fit to the data of the two groups (Hair et al., 2009).

**Table 4:** Model fit information.

Fit indices	Benchmark value	Result	
		Children	Adults
*x*^2^/df	<3	2.326	2.242
RMSEA	<0.08	0.059	0.061
NFI	>0.9	0.904	0.932
GFI	>0.9	0.912	0.907
AGFI	>0.8	0.878	0.871
CFI	>0.9	0.942	0.961

**Figure 3:**
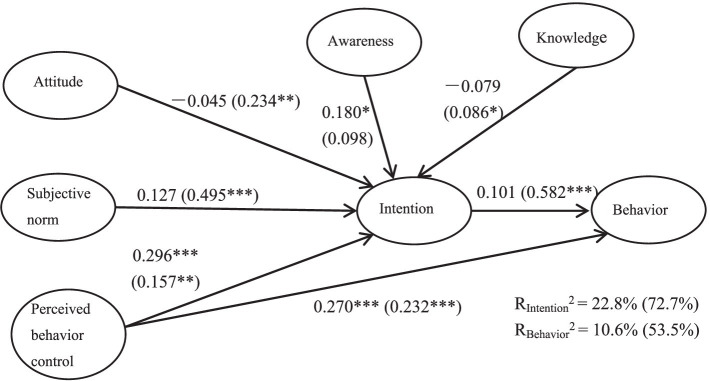
Results of structural equation model. The values inside the parentheses indicate the standardized coefficients for adults. The values outside the parentheses indicate the standardized coefficients for children. *p < 0.05, **p < 0.01, and ***p < 0.001.

Figure 3 shows the standardized coefficients of each hypothetical path in the model of children. Data indicates that children’s perceived behavioral control and waste separation awareness had a significantly positively impact on waste separation intention. Meanwhile, Perceived behavioral control also had a direct positive and significant impact on waste separation behavior. Thus, H1.3, H1.4, and H3 were accepted.

Children’s waste separation attitude, subjective norm, and knowledge had no significant impact on waste separation intention, and waste separation intention had no significant impact on behavior. H1.1, H1.2, H1.5, and H2 were rejected.

While comparing the two groups, several differences were observed. First, the *R*^2^ values of children’s and adults’ waste separation intention were 22.8 and 72.7%, respectively. The *R*^2^ values of children’s and adults’ waste separation behavior were 10.6 and 53.5%, respectively. It can be conclude that the model’s power to explain children’s waste separation intention and behavior was lower than that to adults. Thus, H4 was confirmed.

Second, most path of adults’ model reached a significant level, except for awareness. While only three paths (perceived behavioral control to intention, awareness to intention, and perceived behavioral control to behavior) were significant for children’s model.

## 5 Discussion

The present study provides an expanded model of TPB to explore 9 to 12 years old children’s waste separation behavior, and makes a comparison between the key factors of children’s and adults’ waste separation behavior. Environmental behavior in childhood has a significant positive effect on individual’s such behavior in adulthood (Evans et al., 2007; Arı and Yılmaz, 2017; Otto et al., 2019), and can affect the environmental attitude and behavior of elders around them (Meinhold and Malkus, 2005; Liao and Li, 2019; Wang et al., 2022). Thus, promoting children’s environmental behavior could benefit both current and future environmental quality. While numerous previous studies on waste separation have focused on adults, few studies have paid attention to children. Thus, this study is one of the pioneer attempts to use an expanded TPB model to understand children’s waste separation behaviors and how they differ from those of adults. Several findings can be drawn from the study.

First, we found a paradoxical result when comparing the scores of the scales between children and adults. For the six latent variables (attitude, subjective norm, perceived behavioral control, awareness, intention, and behavior), children’s means were higher than adults. Interestingly, when came to the objective items of knowledge, the result was just the opposite, children’s correct percentage was lower than adults’. According to prior studies, this may be due to social desirability bias. When responding to self-report questions, children are more vulnerable to such effects than adults (Baxter et al., 2004; Logan et al., 2008; Krumpal, 2013; Camerini and Schulz, 2017). Therefore, when answering the self-report questions about waste separation, children would more prone to take into account social desirability, thus reporting higher scores than adults. Meanwhile, some other studies claimed that 9–13 years old was the stage when people would show the highest environmental value and behavior (Collado et al., 2015), which may be another reason for such a result. As for the waste separation knowledge, adults are the primary force separating waste in a family, children have fewer opportunities to participate in waste separation directly and deal with less types of waste than adults (Collado et al., 2015). This may be why children’s objective waste separation knowledge is lower than that of adults. The intention-behavior discrepancy is consistent with numerous studies (Zhang et al., 2019; Wang et al., 2020), and behavior can be influenced by many contextual factors in addition to subjective intention.

Second, as for the TPB variable, the only significant factor influencing children’s waste separation intention and behavior was perceived behavioral control. This result is consistent with the original TPB model (Ajzen, 1991) and many other previous studies (Chan and Bishop, 2013; de Leeuw et al., 2015; Goh et al., 2022). That is, children are more likely to engage in waste separation behavior when they feel facilitated or capable of doing so. Surprisingly, attitude and subjective norm were found to be non-significant predictor of waste separation intention and behavior, and waste separation intention also failed to predict the behavior.

Attitude was found not a reliable predictor of behavior in other studies too. Numerous studies confirmed that attitude can positively influence environmental intention (de Leeuw et al., 2015; Kumar, 2019; Zhang et al., 2019; Wang et al., 2020), while others argued that a stronger attitude may not always be leading to stronger intention (Collado et al., 2015, 2017; Casaló and Escario, 2018; Otto et al., 2019; Bechler et al., 2021; Šorytė and Pakalniškienė, 2021). Bechler et al. (2021) noted that attitude would have an effect on behavior only when it crosses the valence threshold (from positive to negative or vice versa). According to Casaló and Escario (2018), children may not have enough opportunities to engage in certain environmental behaviors, which could reinforce or weaken the translation from attitude to willingness of behavior. Collado et al. (2017) also noted that older groups are more likely than younger groups to translate their attitude into behavior.

Similarly, subjective norm being non-significant in this study is not unique. While Ajzen (1991) argued that social pressure is an important antecedent for subjective norm to work, subjective norm does not always lead to social pressure, especially in the private behavioral domain where others are invisible (Ma et al., 2018; Zhang et al., 2019; Alzubaidi et al., 2021; Yang et al., 2021), such as waste separation behavior. Furthermore, prior studies have found that children over the age of 13 are more sensitive to social pressure (Yeager et al., 2018), our samples may be not mature enough to consider what others prefer them to do.

Third, the role of two expanded variables, awareness and knowledge, in promoting waste separation intention were explored. In line with many previous studies, the greater the awareness of the consequences of not separating waste, the greater the intention (Zhang et al., 2019; Bülbül et al., 2020; Goh et al., 2022; Oturai et al., 2022). Individuals who are aware of the potentially harmful consequences of not separating waste are more concerned about their environmental behavior, thus, more likely to change their behavior mode to mitigate the harm (Zhang et al., 2019; Bülbül et al., 2020; Oturai et al., 2022). When come to the relationship between knowledge and waste separation intention, the findings of prior studies are divergent. Some researchers found a positive relationship (Pagiaslis and Krontalis, 2014; Zhang et al., 2017; Casaló et al., 2019; Cologna et al., 2022) while others disagree with these findings (Grob, 1995; de Leeuw et al., 2015; Otto et al., 2015; Otto and Pensini, 2017; Taube et al., 2021). The non-significant relationship in this study may be due to the fact that the process of knowledge acquisition is not accompanied by the action and the real environment (Otto and Pensini, 2017; Liu et al., 2020). Furthermore, simply focusing on objective knowledge could be another reason for the non-significant results. Pagiaslis and Krontalis (2014) found that subjective knowledge, rather than objective knowledge, had a significant impact on the intention to pay higher prices for eco-fuels. Similarly, Casaló et al. (2019) found that people with high subjective knowledge were more likely to engage in daily environmental behaviors.

Finally, several differences were found by comparing the path coefficients and the percentage of explained variance of the two groups. Adults’ explained variance of waste separation intention and behavior (*R*_Intention_^2^ = 72.7%; *R*_Behavior_^2^ = 53.5%) were greater than those of children (*R*_Intention_^2^ = 22.8%; *R*_Behavior_^2^ = 10.6%). Furthermore, unlike children, adults’ attitude, subjective norm, and perceived behavioral control all had a significant impact on their waste separation intention, and perceived behavioral control and intention can both had a significant impact on their waste separation behavior. This could be due to both the fewer opportunities for children to engage in waste separation practice directly, and their inability to accurately predict the consequences of their behavior (Roux et al., 2021; Šorytė and Pakalniškienė, 2021; Jeremic Stojkovic et al., 2022). Furthermore, the role of the two extended variables, awareness and knowledge, in the adults’ model, was contrary to children’s. Adults’ knowledge had a significant positive effect on waste separation intention, whereas awareness did not. Previous studies have confirmed the effect of knowledge (Meinhold and Malkus, 2005; Pagiaslis and Krontalis, 2014; Pothitou et al., 2016; Liao and Li, 2019), and the non-significant relationship between awareness and intention may be due to the lack of relevant information (Liobikienė and Juknys, 2016). Meanwhile, Fu et al. (2020) argued that situational factors have a greater impact on intention than internal motivations such as awareness.

## 6 Conclusion

Children are not only the important force of current waste separation but also the main drivers and policymakers of the future. Meanwhile, they can influence the waste separation behavior of adults. Hence, there is a strong need to study the factors influencing children’s waste separation behavior. To our knowledge, the present study is one of the few studies to employ an expanded model of TPB to explore children’s waste separation behavior. This expanded model takes into account knowledge and awareness in addition to origin measures of attitude, subjective norm, and perceived behavioral control as predictors of waste separation intention and self-reported behavior. Furthermore, the findings of this study have helped create a better understanding of the differences between children’s and adults’ waste separation behavior.

The study made a number of implications for environmental course developers and policymakers. First, perceived behavioral control is proved crucial to enhance children’s waste separation intention and behavior. Therefore, environmental agencies should take steps to increase their perceptions of how easy it is to engage in waste separation behavior, such as lowering the trash bins, making the bins more visible, positioning the bins closer to homes or classrooms, and placing waste separation guidance near the bins. Second, children’s waste separation knowledge is significantly lower than that of adults. Although the role of knowledge on waste separation intention is not proven, however, it is impossible to adopt the proper behavior without action-oriented knowledge. Therefore, environmental organizations and educational institutions should provide procedural knowledge to children in various ways, such as the internet and training courses. Moreover, it is better to incorporate real-world applications into the knowledge transfer process. Third, given that children’s explained variance of intention and behavior are lower than those of adults, furthermore, unlike adults, children’s intention was found to have no significant impact on behavior, schools should focus more on promoting children’s cognitive abilities such as decision making and consequence prediction. Finally, to narrow the gap between waste separation behavior and intention, authorities should provide more external conditions that facilitate waste separation for both children and adults, such as financial incentives and mandatory rules.

There are some limitations about the present study that future research can address. First, we did not include children/adolescents aged under 9 years old or 12–18 years old. Due to method and resource constraints, we decided to focus on mid-age children who are 9–12 years old to determine the factors influencing children’s waste separation behavior and explore the differences with adults. Future studies could expand the age range to explore the development patterns of waste separation behavior. Second, the study explored correlations between variables, and future research could explore causality by manipulating variables. In addition, this study collected data through self-reports and therefor may have been influenced by socially desirable answers. Incorporating objectively measured behaviors by observing and recording actual behaviors would be beneficial for future studies.

## Data availability statement

The original contributions presented in the study are included in the article/supplementary material, further inquiries can be directed to the corresponding author.

## Ethics statement

The studies involving humans were approved by Institutional Review Board of the Institute of Psychology, Chinese Academy of Sciences. The studies were conducted in accordance with the local legislation and institutional requirements. Written informed consent for participation in this study was provided by the participants’ legal guardians/next of kin. Written informed consent was obtained from the individual(s) for the publication of any potentially identifiable images or data included in this article.

## Author contributions

JP: Conceptualization, Data curation, Formal analysis, Investigation, Methodology, Software, Writing – original draft, Writing – review & editing. PL: Methodology, Project administration, Supervision, Validation, Writing – review & editing.
